# The Plans and Purposes of the Johns Hopkins Hospital.—I


**Published:** 1889-08-17

**Authors:** John S. Billings

**Affiliations:** Surgeon, U.S. Army


### The Plans and Purposes of the Johns Hopkins Hospital.—I.*

By John S. Billings, M.D., Surgeon U. S. Army.

### I.—INSTRUCTIONS AND CARDINAL PRINCIPLES.

The third paragraph of the letter of instructions communicated by Johns Hopkins to the trustees whom he had chosen to carry out his plans for a hospital in the city of Baltimore states that "it is my wish that the plan . . . shall provide for a hospital which shall, in structure and arrangement, compare favourably with any other institution of like character in this country or in Europe." What do you suppose the writer was thinking of when he penned that sentence? Had he in view any definite ideal, any mental picture of the institution which he proposed to establish, or was it merely an expression of a desire to give to his city the best thing that could be devised? I have read that letter many times, have heard much of the ideas, hopes, and wishes which were expressed in the numerous conversations which preceded its preparation, and it seems to me that the writer had an ideal, and not a mere vague desire—an ideal which was no doubt somewhat misty, but which did not correspond to any existing hospital, and one which he did not attempt to define except in a few prominent points to which I shall presently refer. In most respects Johns Hopkins took the same course with his hospital which he did with his university, and deliberately refused to trammel with specific directions those whom he had chosen to carry out his plans; but this letter of instructions indicates, nevertheless, a conception of much more definite character, and one which had been the subject of more discussion and reflection than his scheme for a university. Whether this be so or not, I am at all events sure that his trustees have endeavoured to comply with this letter of instructions, and to do so in the broadest and best sense of the words.

The beginning of the results we have before us to-day; results which even now are not confined to these particular aggregations of bricks and mortar, as will be presently explained, and the end of which will be, as we hope and believe, to make life happier for millions now living and yet unborn. Only those who took part in the early deliberations of those charged with this trust can fully realise the anxieties, the doubts, the manifold perplexities which at first attended their decisions and movements. Only one or two of them had any knowledge of hospital matters; most of them were business men, bankers, lawyers, judges, railway managers, men who knew something of the management of men and money, but who were now brought face to face with a new problem—viz., how to build, organise, and manage a hospital so that it should compare favourably with any other hospital in this country or in Europe.

To "compare favourably with," what does that mean? It is a peculiar phrase, which, coming from a shrewd business man and a member of the Society of Friends, signifies, I think, to excel, if possible; at all events, that is the safest interpretation. And it was not this or that hospital which was to be surpassed or equalled, but all other hospitals in this country or in Europe; Africa, Asia, and Australasia being put out of the question. It was a large contract.

The location was fixed—that had been done by Johns Hopkins—but they had to decide whether the structures to be erected should be temporary or permanent, of wood, brick, or marble, in one large building or in many, and many other like points, before even the preparation of plans could be commenced. They followed the instructions of the donor, and got advice, of which a great abundance was available. They visited the large hospitals of our Eastern cities, employed five men, supposed to be skilled in hospitals, each to write an essay giving his plans and suggestions, published these essays in a book which had a wide circulation, and studied the criticisms and reviews to which this book gave rise.

Having duly considered the multifarious and widely-divergent suggestions thus obtained, they finally selected one of the essay-writers, and asked him if he was satisfied with his own plans now that he had seen the others and the published criticisms upon them. He promptly said that he was not, whereupon they asked him to try again and do better. He set to work, aided by the architect of the board, and the result was a set of sketch plans which he took abroad and obtained much counsel and criticism on, examining at the same time the model hospitals of Europe. He was much less satisfied with tbe sketch plans when he came back than he was when he started, and again the building committee, the architect, and himself reviewed the whole matter, and finally settled on the general arrangement which you will see to-day. Many details remained to be worked out. Even the façades had not yet been designed; but the general scheme was settled, and the rest was comparatively easy for the time being.

Let us now, for a few moments, consider the broad general principles which governed the trustees in the adoption of this plan. The first hospitals were established to give shelter and food to the sick poor, especially those who gather in cities. Gradually physicians found that they could learn much in these aggregations of suffering, and that they afforded means of teaching others; but this last use of them is only about two hundred years old. Gradually, also, it came to be known that the knowledge thus obtained in the care of the sick poor was of use in treating the diseases of the well-to-do; and, finally, within the last twenty-five years or so, people are beginning to find out that when they are afflicted with certain forms of disease or injury they can be better treated in a properly appointed hospital than they can be in their own homes, no matter how costly or luxurious these may be. In the hospital they can have not only all the comforts of home, but more; not only skilled medical attendance and skilled nursing, but the use of many appliances and arrangements specially devised for the comfort and welfare of the sick which can hardly be found in any private house, and also freedom from noise and many petty annoyances, including in some cases too much sympathy and in others too little. This hospital, then, is to provide for the rich as well as for the poor; for those who can, and ought to pay for the help given, as well as for those who cannot.

A second cardinal principle to be observed in such a hospital as this, is that it shall do as little harm as possible. A hospital may do harm by its foul air, by spreading contagious disease among its inmates, by neglect or carelessness of its nurses or attendants; and in years gone by hospitals have, no doubt, caused nearly as much sickness as they have relieved. This is now rarely the case, and in this hospital the arrangements for ventilation, isolation, and nursing are such as to entirely do away with this danger. There is another danger connected with free hospitals and dispensaries which is of quite a different kind, and to which I can here only allude—namely, the danger of promoting negligence, shiftlessness, laziness, and vice by offering free relief from their consequences—the danger of pauperising people. This is a danger connected with organisation and management rather than with construction, and I can only say here that it has been foreseen, and will be, as far as possible, guarded against.

* An address delivered at the opening of the hospital, May 7, 1889.

The third principle to be kept in view in such a hospital as this, is that it should provide the means of giving medical instruction, for the sake of the sick in the institution as well as of those out of it. It is well known to those familiar with the subject that the sick in a hospital where medical instruction is given receive more constant, careful, and thoughtful attention than do those in a hospital where no such instruction is given. The clinical teacher must do his best; keen eyes will note every error in diagnosis, every failure in results of treatment. Moreover, the very act of teaching clarifies and crystallises his own knowledge; in attempting to explain, the dark places become prominent and demand investigation, and hence it is that those cases which are lectured on receive the best treatment. I need say nothing here on the other side of the question, the value of properly trained physicians to the community and the necessity for hospital instruction in such training. Johns Hopkins understood all this, and specially directed that "in all your arrangements in relation to this hospital you will bear constantly in mind that it is my wish and purpose that the institution shall ultimately form a part of the medical school of the university."

Now there are medical schools and medical schools, and in obeying this direction of the donor the trustees had to consider what sort of a medical school this school of the university was likely to be. As the majority of the trustees were also trustees of the university, they knew well the principles which underlie the organisation of that institution, and that the same principles would govern the organisation of the medical department when that came to be taken in hand. One of these principles is the thorough teaching of that which is known, another is to increase that which is known, and to furnish the men and means for doing this. So also the hospital should not only teach the best methods of caring for the sick now known, but aim to increase knowledge, and thus benefit the whole world by its diffusion. Another point which had to be kept in view was the direction of Mr. Hopkins that there should be established, "in connection with the hospital, a training school for female nurses, not only to care for the sick in the hospital, but to benefit the whole community by supplying it with a class of trained and experienced nurses."

It is also highly desirable that a hospital of this kind should have connected with it a well-appointed dispensary for the treatment of those who need medical aid but not a bed in the hospital. Through such a dispensary much good can be done at small cost, the selection of proper patients for the hospital is facilitated, the means of medical investigation and teaching are greatly extended, and the scope of the nursing system can be made to reach the poor and ignorant in their own homes.

*(To be continued.)*

